# Cross-hemispheric Alternating Current Stimulation During a Nap Disrupts Slow Wave Activity and Associated Memory Consolidation

**DOI:** 10.1016/j.brs.2014.12.010

**Published:** 2015

**Authors:** Peter Garside, Joseph Arizpe, Chi-Ieong Lau, Crystal Goh, Vincent Walsh

**Affiliations:** a Institute of Cognitive Neuroscience, University College London, 17 Queen Square, London WC1N 3AR, UK; b University College London Medical School, Gower Street, London WC1E 6BT, UK; c National Institute of Mental Health, National Institutes of Health, 9000 Rockville Pike, Bethesda, MD 20892, USA; d Department of Neurology, Shin Kong Wu Ho-Su Memorial Hospital, Taipei, Taiwan

**Keywords:** Memory consolidation, Declarative memory, tACS, Sleep, Nap, Montage, Slow wave sleep, Delta wave, Oscillations

## Abstract

**Background:**

Slow Wave Activity (SWA), the low frequency (<4 Hz) oscillations that characterize Slow Wave Sleep (SWS) are thought to relate causally to declarative memory consolidation during nocturnal sleep. Evidence is conflicting relating SWA to memory consolidation during nap however.

**Objective/hypothesis:**

We applied transcranial alternating current stimulation (tACS) – which, with a cross-hemispheric electrode montage (F3 and F4 – International 10:20 EEG system), is able to disrupt brain oscillations–to determine if disruption of low frequency oscillation generation during afternoon nap is causally related to disruption in declarative memory consolidation.

**Methods:**

Eight human subjects each participated in stimulation and sham nap sessions. A verbal paired associate learning (PAL) task measured memory changes. During each nap period, five 5-min stimulation (0.75 Hz cross-hemispheric frontal tACS) or sham intervals were applied with 1-min post-stimulation intervals (PSI's). Spectral EEG power for Slow (0.7–0.8 Hz), Delta (1.0–4.0 Hz), Theta (4.0–8.0 Hz), Alpha (8.0–12.0 Hz), and Spindle-range (12.0–14.0) frequencies was analyzed during the 1-min preceding the onset of stimulation and the 1-min PSI's.

**Results:**

As hypothesized, power reduction due to stimulation positively correlated with reduction in word-pair recall post-nap specifically for Slow (*P* < 0.0022) and Delta (*P* < 0.037) frequency bands.

**Conclusions:**

These results provide preliminary evidence suggesting a causal and specific role of SWA in declarative memory consolidation during nap.

## Highlights

• Cross-hemispheric tACS disrupted low frequency oscillation generation in afternoon nap.
• Oscillation disruption correlated with declarative memory consolidation disruption.
• Absent stimulation, low frequency oscillations also correlated with consolidation.
• A rebound in low frequency oscillations was observed after 29 min of stimulation.
• A causal role of low frequency oscillations in consolidation during nap is suggested.

## Introduction

Memory consolidation is a process whereby new memories are integrated into a pre-existing stable network of long-term associations [1]. Consolidation is strongest during ‘off-line’ periods when there is no interference from new encoding-such as during sleep [2], [3], [4]. Several studies report the importance of slow wave sleep (SWS) in the consolidation of declarative, consciously accessible memories [5], [6], [7]. It is believed that during SWS, slow oscillations temporally coordinate hippocampal and thalamic brain activity during the depolarizing up-state of the oscillation. This hippocampal-neocortical dialog is thought to underly the transfer of information between brain structures and their memory systems [8], [9].

Most studies investigating sleep and declarative memory have focused on effects of a full night of sleep rather than an afternoon nap. Afternoon naps occur under reduced homeostatic sleep pressure and less advanced circadian phase relative to early nocturnal sleep, both of which are known to influence sleep's electrophysiological profile [10]. Thus it is unclear whether results from full night sleep studies generalize to afternoon nap. Only a small number of published studies have investigated whether a daytime nap is sufficient for declarative memory consolidation. One study opposes [11], whilst four studies support this notion. Of those in support, two found consolidation related to SWS [12], [13] and two report no correlation [14], [15]. Our study aimed to clarify this relationship.

Further, we sought to determine if slow wave activity (SWA) – slow oscillatory (0.7–0.8 Hz) and delta activity (1.0–4.0 Hz) – has a causal influence on declarative memory consolidation by measuring memory changes resulting from disruption of SWA. A small number of full night sleep studies have already provided evidence of this causal relation [16], [17], [18]. Marshall and colleagues effected an increase in SWA during non-REM (NREM) sleep by using bilateral frontolateral tDCS to intermittent slow-oscillation-like (0.75 Hz) potential fields through the cortex. They enhanced retention of the word-pair associations. Stimulation was only delivered during the first period of NREM sleep with no effect during the remainder of the night. It is reasonable then to hypothesize that manipulation of SWA during an afternoon nap would also affect declarative memory.

**Figure 1:**
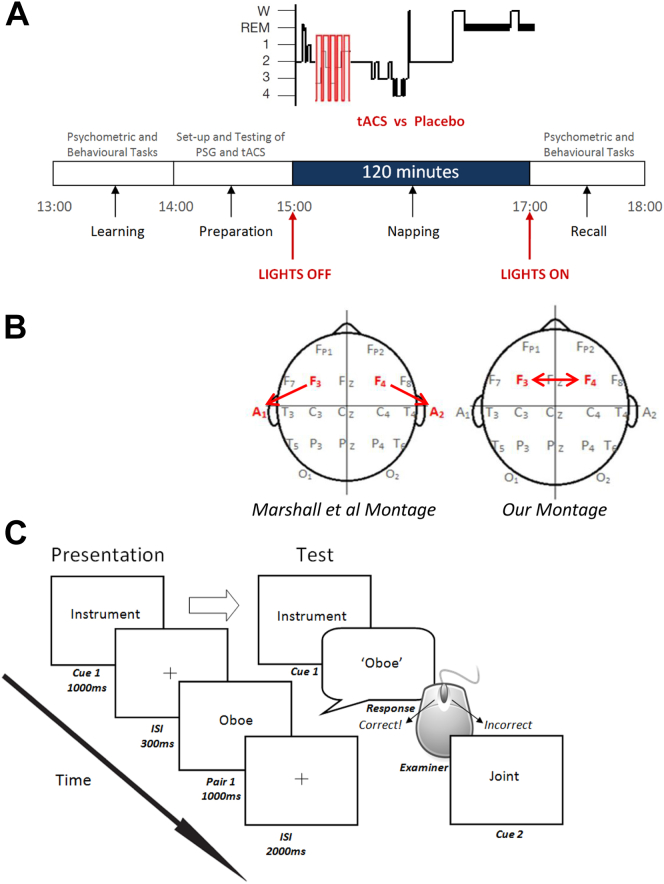
Experimental set up. A, Experimental design. B, Electrode montage. C, PAL task protocol: Following initial presentation and testing (as shown in the diagram) if subjects scored > 30% correct, they progressed to the preparation stage (see Fig. 1A). If however, they scored <30% correct then they were presented the word list again at twice the presentation speed. Following representation they were again tested without feedback, as before. No subjects failed to score 30% following the second presentation.

We delivered sinusoidal tACS, which is able to entrain [19], [20] and also hypothesized to be able to desynchronize [21], [22] neuronal oscillations. Oscillations are often generated by two symmetrically located neural generators, one in each hemisphere [23], [24], [25], [26], and modeling suggests that cross-hemispheric sinusoidal tACS can disrupt neural functions governed by inter-hemispheric phase synchronization [21]. Because slow oscillations originate predominately in the prefrontal cortex [27], we targeted this area with cross-hemispheric stimulation (see Fig. 1b). We hypothesized that sinusoidal cross-hemispheric frontal tACS would disrupt slow oscillation generation, inhibiting SWA and memory consolidation.

## Materials and methods

### Participants

Eight subjects (4 female) aged 20–22 (mean 21 ± 0.926) participated in both experimental sessions. All provided written informed consent, and the University College London ethics committee approved all experimental procedures. All participants were fluent English-speaking students enrolled at University College London. Subjects were recruited who reported being capable of afternoon nap and no history of neurological, psychiatric or sleep disorders, drug or alcohol abuse.

### Experimental design

Subjects were instructed to avoid caffeine, alcohol, and psychoactive substances for 12 h prior to experimentation. Each subject participated in two sessions: a stimulation session and a sham stimulation (control) session (Fig. 1A). The order of stimulation/sham and of word list version was counterbalanced across the eight subjects, who were naïve to which session they received stimulation.

To control for circadian and homeostatic factors affecting sleep architecture [3], testing always began at 13:00. Two standard psychometric tests (the Weschler Adult Intelligence Scale Digit Span Test and a word fluency task, see *Psychometric tests* in Supplementary Methods) were carried out to assess general retrieval function, wakefulness and working memory. Following these tests, subjects carried out training and pre-nap testing for a Paired Associate Learning (PAL) task (Fig. 1C, see Supplementary Methods), which served as a measure of declarative memory. Then EEG electrodes were attached and polysomnographic recording was set up and tested.

At approximately 15:00, subjects were instructed to nap for a 120-min period in a dark room. To control for effects of sleep inertia, if a subject completed a sleep cycle near the end of the nap opportunity, the subject was woken before they entered a further cycle, and if a subject was in deep (stage 3 or 4) sleep at the 2 h mark, they were not woken until they re-entered light sleep. During stimulation sessions, subjects underwent five stimulation periods, each 5 min in duration, followed by 1-min inter-stimulation intervals that were stimulation free, totaling 25 min of stimulation over a 30 min period. Stimulation always began eight epochs (30 s per epoch) after subjects had entered NREM sleep stage-2 without any transition back to NREM sleep stage-1 or stage-Wake. During sham sessions, no stimulation was delivered during the nap.

Subjects were woken around 17:00 (depending on their sleep cycle), and given a short time to wash and rehydrate before the two psychometric tests were performed again. If a subject scored lower than pre-nap on these tests, they were re-tested until performance equivalent to pre-nap was reached so as to equate cognitive performance pre- and post-nap before assessing memory recall. Following this, subjects were re-tested on the PAL task they had undertaken before the nap.

### Polysomnographic (PSG) recording

See Supplementary Methods.

### Electrical stimulation

Transcranial alternating current stimulation (tACS) was applied as a 0.75 Hz bipolar sinus wave (550 μA maximum amplitude). A battery operated DC-Stimulator Plus (NeuroConn. Ilmenau, Germany) delivered the current to subjects via two conductive rubber electrodes (20  mm × 25  mm) at F3 and F4 attached to the scalp with the same conductive, adhesive wax used for the PSG electrodes (Fig. 1B). Wax was used in preference of saline-soaked sponges as it would not be possible to reapply saline during a nap session without waking the subject. Our maximum current density matched the high end of currently establish protocols [28], and total current approximated that used in the study by Marshall and colleagues [16]. Thus, with our 5 cm^2^ electrodes, the current density was 110 μA/cm^2^.

Subjects were blind to which session was sham as electrodes were attached and comfort and impedance testing occurred in both sessions. To reduce the likelihood of the subject waking up, ten amplitude-graduated wind-up and wind-down cycles were used on initiation and termination of the stimulation, respectively. Our subjects did not report pain during the sessions, and stimulation parameters were within safe limits of duration and intensity, not exceeding tested protocols [28], [29].

### Analyses

Percentage accuracy was recorded for the word-pair task before and after nap, and the difference was calculated for each session. EEG data were processed with custom Matlab (7.9.0 R2009b, Mathworks) scripts using the EEGLAB toolbox [30] (http://www.sccn.ucsd.edu/eeglab/) and Fieldtrip [31] (http://fieldtrip.fcdonders.nl/) libraries to estimate spectral power. Slow (0.7–0.8 Hz), delta (1.0–4.0 Hz), theta (4.0–8.0 Hz), alpha (8.0–12.0 Hz), and spindle-range (12.0–14.0 Hz) frequency band spectral densities for each interval of interest were estimated using a Hanning taper method of spectral estimation (‘mtmconvol’ spectral calculation method in Fieldtrip, with taper set to hanning). Several contiguous frequency bins (the number of bins per Hz in each frequency band were equated: 5 for slow, 61 for delta, 81 for theta and alpha, and 41 for spindle-range; the width of each bin were equal on a log scale, specifically, a given bin width = e^iw^ − e^(i − 1)w^, where *w* = [log(freq_windowMax_) − log(freq_WindowMin_)]/*n*, *n* = number of bins, and *i* = ith bin) were estimated in each frequency band. Data series (per channel) were segmented into Hanning windows 30 s wide, each centered 5 s apart, but not overlapping with the stimulation periods. Resulting spectral power values for each frequency band were averaged across channels (C3, C4, and Fz), frequency bins, and Hanning time windows. We report power in units of μV^2^/Hz. For calculating correlations between EEG frequency bands and recall performance in the sham stimulation condition, spectral power during the entire nap period was used. Arousals were manually spliced out from the original EEG traces in order to remove spikes from the spectral data before further analysis was carried out. When determining the effect of stimulation on EEG oscillations, the 1-min periods following the five stimulation or corresponding sham periods, in addition to the 1-min period before the first stimulation or sham period, were used as the intervals of interest. Power in each pre-stimulation interval was taken as baseline for the given nap session and so was subtracted from the power of each of the post-stimulation intervals to calculate the power index of each post-stimulation interval of each subject. When correlating changes in spectral power (stimulation relative to sham) to changes in subject performance on the PAL task, spectral power was calculated as the average of all five post-stimulation intervals. In addition to arousals, other artifacts (EEG electrode pops and muscle contraction artifacts) were removed from each 1-min post-stimulation interval before analysis. Online visual scoring of sleep stage according to Rechtschaffen and Kales [32] criteria determined initiation of stimulation. A researcher who was involved neither in the data collection nor in the spectral analyses also later visually scored offline all sleep traces in 30-s epochs according to Rechtschaffen and Kales [32] criteria, and these data were subsequently analyzed (see Supplementary Methods and Results). We refrained from utilizing Rechtschaffen and Kales style [32] visual sleep stage scoring in our main analyses because our spectral analysis allowed for a much more objective and quantitative measure of specific types of oscillations of interest than visual scoring.

Throughout the analyses, student's *t* and Pearson's product moment correlation tests were employed. When possible the statistical tests were performed within subject. Because SWA was hypothesized to relate positively to performance and to be specifically disrupted by the stimulation, tests concerning these relationships for slow and delta frequency bands were conducted one-tailed, otherwise all tests were conducted two-tailed. It is reported where violations of the assumptions of the statistical tests were present (e.g. an outlier driving a correlation).

## Results

### Subject inclusion

One subject was excluded when analyzing the stimulation data as he was unable to sleep for the full duration of stimulation. Another subject's fifth post-stimulation interval in the stimulation condition was excluded from analysis due to an error in stimulation settings.

### Correspondence of sleep time and latencies between stimulation and sham sessions

Neither sleep period (time from sleep onset to final awakening; Stim: Avg = 112.3 min, StDev = 9.7 min. Sham; Avg = 103.7 min, StDev = 25.8 min. Paired *t*(6) < 0.90, *P* > 0.40 two-tailed) nor sleep latency (Stim: Avg = 5.00 min, StDev = 3.6 min. Sham: Avg = 9.6 min, StDev = 15.1 min. Paired t(6) < 1.02, *P* > 0.35 two-tailed) significantly differed between stimulation and sham sessions, suggesting that the two conditions were sufficiently matched on these variables. The stimulation artifact precluded total sleep time comparison. Other sleep variables are presented in Supplementary Results.

### Alignment of stimulation and sham pre-stimulation intervals

Mean power in the frequency bands (slow, delta, theta, alpha and spindle-range) was calculated for each of the six 1-min intervals flanking the five stimulation or sham intervals (see Methods section). Pre-stimulation 1-min interval power values did not differ between stimulation and sham conditions (*t*(6) < 1.315, *P* > 0.23 two-tailed for all frequency bands, *P* > 0.558 for both slow and delta bands) suggesting that PSIs were well matched between the stimulation and sham conditions.

### PAL task and SWA

**Figure 2:**
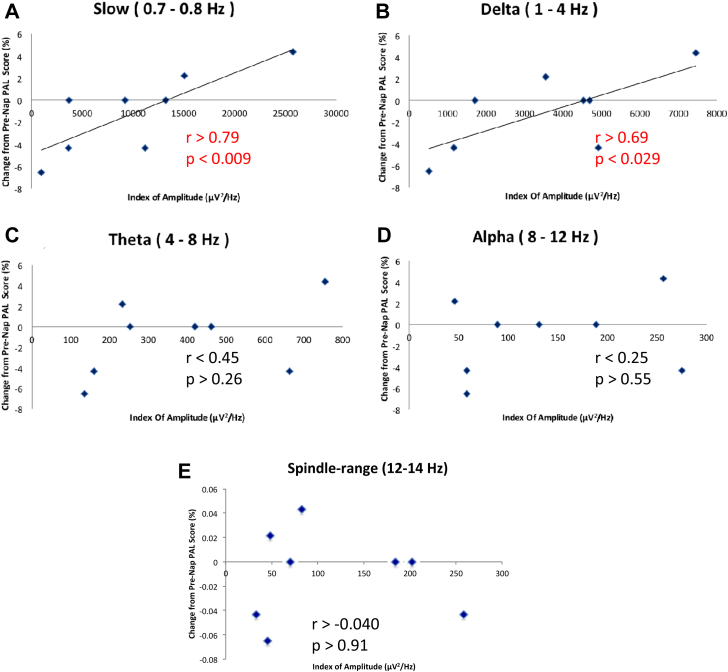
Slow and delta power of slow wave sleep specifically correlate with declarative memory consolidation during afternoon nap. Correlations between subject performance in the PAL task and subject total power value over the entire sham sleep interval for each spectral bands – slow, delta, theta and alpha – during the nap period. A significant positive correlation was found between the PAL task and slow and delta bands [Pearson's correlation; A, *r* > 0.79, *P* < 0.009, one-tailed, B, *r* > 0.69, *P* < 0.029, one-tailed], but not with theta, alpha, or spindle-range bands [C, *r* < 0.45, *P* > 0.26, two-tailed, D, *r* < 0.25, *P* > 0.55, two-tailed, E, *r* > −0.040, *P* > 0.91, two-tailed].

For the sham condition, a significant positive correlation was observed between frequency band power density and PAL task performance change over the nap interval for slow (*r* > 0.79, *P* < 0.0099 one-tailed) and delta (*r* > 0.69, *P* < 0.029 one-tailed) frequency bands, but not for the theta, alpha (both *r* < 0.5, *P* > 0.26, two-tailed), or spindle-range (*r* > −0.040, *P* > 0.91) bands (Fig. 2).

### PAL task and stimulation

**Figure 3:**
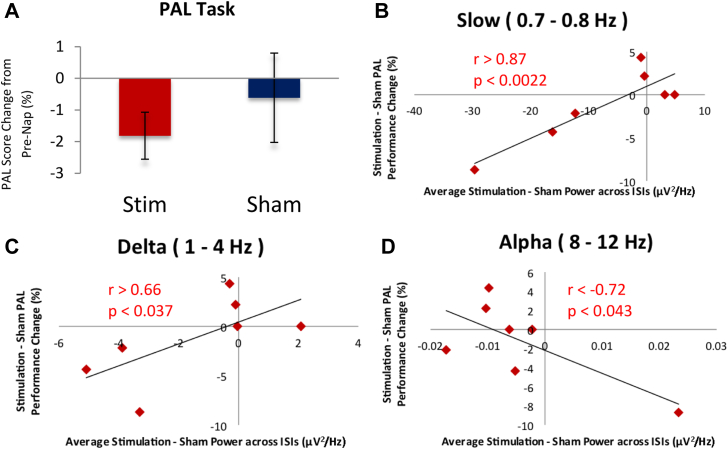
–Effect of stimulation on memory consolidation. A, Overall effect of stimulation on PAL performance change after nap was not found to be statistically significant. Paired *t*(6) < 0.77, *P* > 0.23, one-tailed. Error bars indicate standard error of the mean. Scatterplots correlating change in baseline spectral power due to stimulation with change in PAL performance due to stimulation for B, Slow; C, Delta; and D, Alpha frequency bands.

Our stimulation paradigm seems to have impaired memory consolidation. Average decrement in PAL performance was numerically of greater magnitude with stimulation than sham. There was no significant difference between these decrements (paired *t*(6) <0.77, *P* > 0.23 one-tailed) (Fig. 3A); however, as would be expected, the degree to which our stimulation paradigm interfered with the generation of slow and delta oscillations relative to sham correlated with the degree of impairment on the PAL task relative to sham (*r* > 0.877, *P* < 0.0022 one-tailed for slow and *r* > 0.663, *P* < 0.037 one-tailed for delta) (Fig. 3B and C). Conversely, change in alpha band power between stimulation and sham was negatively correlated with change in performance (*r* < −0.72, *P* < 0.043 two-tailed) (Fig. 3D), though this may be driven by the influence of one data point. No other frequency band produced a significant correlation (both *r* > −0.51, *P* > 0.19 two-tailed). These results suggest that slow and delta oscillations are selectively and causally involved in declarative memory consolidation during nap.

### Time course and frequency specificity of the effects of stimulation

**Figure 4:**
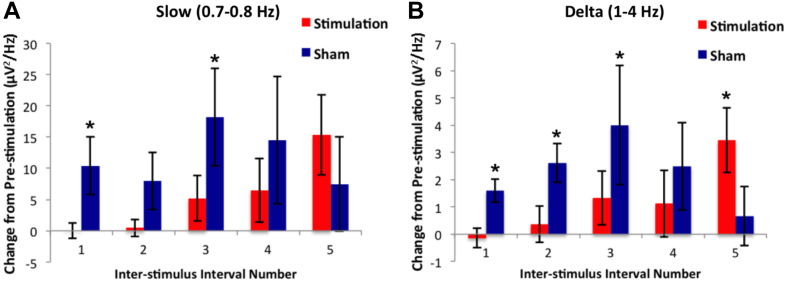
Suppression and Rebound in Slow and Delta Power following stimulation. Change in Slow (0.7–0.8 Hz) and Delta (1–4 Hz) spectral power relative to baseline (pre-stimulation), in each post-stimulation interval, averaged across subjects and channels (Fz, C3 and C4) for the stimulation (red) and sham (blue) sessions. Error bars indicate standard error of the mean. *P< 0.05. (For interpretation of the references to color in this figure legend, the reader is referred to the web version of this article.)

We next investigated in more depth the time course and frequency specificity of the effects of our stimulation on the electrophysiological profile during nap. In the stimulation condition, PSIs 1–4 each did not significantly differ from pre-stimulation interval power values for both slow and delta bands (all t(6) < 1.47, *P* > 0.19 two-tailed), whereas those of the sham condition were each significantly or marginally higher than pre-stimulation power values (all *t*(6) > 1.97, *P* < 0.049 one-tailed, except for PSI 2 and 4 for slow band and PSI 4 for delta band which were all *t*(6) > 1.42, *P* < 0.103 one-tailed) (Fig. 4A and B). This is consistent with our expectation that our stimulation, but not sham, would interfere with generation of the slow and delta oscillations characteristic of slow wave sleep. However, at PSI 5, delta power in the stimulation condition was significantly higher than pre-stimulation (*t*(5) > 2.9, *P* < 0.035 two-tailed) and slow power was marginally higher (*t*(5) > 2.4, *P* < 0.062 two-tailed), suggesting that there was a rebound of SWA following the fifth stimulation interval (29 min after the start of the first stimulation). At the 5th PSI in the sham condition, slow and delta power were not significantly higher than pre-stimulation values (both *t*(6) < 0.99, *P* > 0.18, one-tailed), suggesting that by that point in the sham condition, subjects were already drifting out of SWS (Fig. 4A and B).

**Figure 5:**
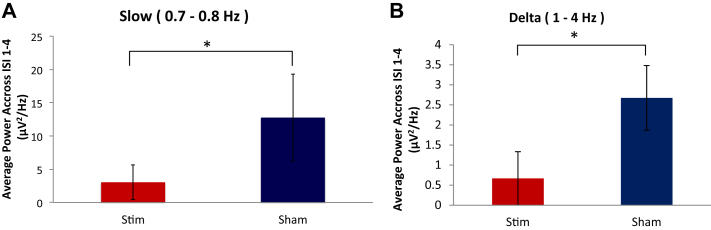
Cross-hemispheric frontal tACS Stimulation Reduces Slow and Delta EEG Power during Afternoon Nap. Change in A, Slow (0.7–0.8 Hz); and B, Delta (1–4 Hz) spectral power compared to pre-stimulation, averaged across subjects, inter-stimulation intervals (1–4 only) and channels (Fz, C3 and C4) for the stimulation and no stimulation sessions. Both slow and delta bands were found to be significant: paired *t*-test, *P* value <0.05. Error bars indicate standard error of the mean.

Change from pre-stimulation power in both the slow and delta bands differed between stimulation and sham for the average of the first four PSIs (both bands *t*(6) < 1.98, *P* < 0.047 one-tailed), but no other frequency band yielded a significant difference (all *t*(6) < 0.81, *P* > 0.44, two-tailed), showing that our stimulation selectively disrupted the slow and delta bands in the first four PSIs (first 24 min) (Fig. 5A and B). Change from pre-stimulation power values did not differ between stimulation and sham conditions in the fifth PSI for any power bands (all *t*(5) < 2.296, *P* > 0.07, two-tailed), though theta and alpha bands yielded marginally lower values for the stimulation condition (both *P* < 0.095). Together these results (Figure 4, Figure 5) suggest that our stimulation paradigm selectively disrupted generation of slow and delta oscillations in the first four PSIs (first 24 min after start of stimulation) (Fig. 5), but that there was a rebound in SWA following the last stimulation period (29 min after start of stimulation) (Fig. 4).

## Discussion

SWA during afternoon nap correlated with declarative memory performance. Cross-hemispheric tACS disrupted the generation of the slow and delta oscillations of SWS and thus appears to have causally disrupted declarative memory consolidation.

### SWA during afternoon nap correlates with declarative memory consolidation

A large body of evidence implicates SWA in the facilitation of declarative memory consolidation during nocturnal sleep [7], [5], [8], [9], [33]. As sleep is a circadian and homeostatic phenomenon, sleep-related memory consolidation may interact with this rhythm. The findings for a relationship between daytime nap and declarative memory consolidation are scarce and contradictory, and there is particular disagreement regarding the relationship of SWS to declarative memory consolidation during nap. For example, Tucker et al. [14] reported that a daytime nap (47 min duration) enhanced performance on a declarative memory task, but found that enhancement did not correlate with SWS. Similarly, Lahl et al. [15] reported enhanced declarative memory retention for both short (less than 6 min duration in which no SWS occurred) and long (60 min) naps compared to wakefulness, but longer naps yielded better memory recall. SWS and memory retention were not correlated, indicating that increased nap time, but not SWS, enhanced consolidation. In contrast, Backhaus and Junghanns [11], who used a comparable total nap time (45 min) to Tucker and colleagues, reported that nap did not significantly enhance post-nap performance, yet found a positive correlation between performance and SWS. Additionally, Schabus et al. [13] reported that a 1-h nap significantly improved declarative memory retention, but only for subjects with SWS. Taken together, three studies support and one opposes the notion that daytime nap improves declarative memory consolidation. Also, two studies suggested that a beneficial effect was related to SWS and two reported no correlation. Our results support the notion that there is indeed a correlation between declarative memory consolidation and SWA during nap. Findings from the whole sleep period in our sham condition revealed a significant positive correlation between memory retention and two frequency bands – classic delta activity (1.0–4.0 Hz) and slow oscillation (0.7–0.8 Hz) (Fig. 2A and B). Power across these frequency bands was used to index the amount of SWA experienced by each subject. The lack of significant correlation between memory and other frequency bands (theta, alpha, and spindle-range) (Fig. 2C–E) suggests specificity in the role of slow oscillation and delta activity in memory consolidation during afternoon nap. Thus, memory consolidation during afternoon nap correlates specifically with the amount of SWA.

### Cross-hemispheric tACS suggests causal role of SWA in declarative memory consolidation during nap

We found additional evidence suggesting that SWA, the slow and delta oscillations characteristic of SWS, are causally and specifically related to declarative memory consolidation during nap. Reduction in memory consolidation in our subjects was strongly correlated with the degree to which stimulation reduced the generation of slow oscillations (Fig. 3B) relative to sham and also significantly correlated with reduction of delta (Fig. 3C), but not theta nor spindle-range, oscillations. Additionally, increase in alpha band power, indicative of lighter (stage 1) sleep, was significantly negatively correlated with memory consolidation (Fig. 3D), though this may have been driven by one subject's datapoint. Together these results suggest that SWA, is causally related to memory consolidation. Our nap study is thus consistent with full-night studies reporting that causal manipulations of SWA induced measurable changes in memory consolidation [16], [17], [18]. In light of this, it may seem surprising that the overall reduction in memory consolidation observed for stimulation compared to sham did not reach significance (Fig. 3A); however, this is likely due to variability across subjects in the degree to which stimulation had an effect on SWA on average across all the inter-stimulation intervals. This variability seemed to be driven by a rebound in SWA after the final stimulation interval, following a period of suppression of SWA. Therefore, we next discuss in more detail the time course of the effect of our stimulation on the electrophysiological profile during nap.

### Cross-hemispheric tACS during nap disrupts slow wave generation, but a rebound follows

Spectral analysis of our EEG data shows that during the first four PSIs – corresponding to the first 24 min from the start of stimulation – selective reduction of both the slow and delta frequency bands occurred (Fig. 5A and B). This interference supports Neuling et al. [21], who modeled current flow during tACS and hypothesized that cross-hemispheric sinusoidal tACS stimulation may disrupt neural functions governed by inter-hemispheric phase synchronization – as seen in frontal brain regions during SWS [34] – as it results in 180° phase shift between the two electrodes [21]. Another potential factor for our disruption of slow waves could be the standard stimulation frequency of 0.75 Hz, which was not tailored to each subject's individual slow wave frequency. Zaehle et al. [20] measured each subject's individual peak alpha frequency prior to stimulation and tailored their stimulation frequency accordingly, augmenting on-going alpha oscillations using a cross-hemispheric electrode montage.

Typically, oscillations are generated by two symmetrically located neural generators, one in each hemisphere [23], [24], [25], [26]. Functional coupling between these generators is reflected by inter-hemispheric phase synchronization [35]. This occurs across low delta (1.0–2.0 Hz), alpha (9.0–10.0 Hz) and spindle (13.0–14.0 Hz) ranges during NREM sleep [36], and predominates anteriorly – in the frontal brain region – during SWS [34]. Inter-hemispheric coherence in delta-range frequencies increases in humans in the transition from wakefulness to sleep [37], and inter-hemispheric EEG correlation in the delta-range has been reported to be higher in stage 2 and stage 4 sleep than in wakefulness [38]. Inter-hemispheric synchrony of EEG oscillations between homologous brain regions of cats were reported to be permanently disrupted when the corpus callosum was sectioned [39], suggesting that connectivity between hemispheres may be functionally relevant. It is not yet clear whether this mechanism underlies slow (0.7–0.8 Hz) oscillations, but our results suggest that it does.

Though the first four PSIs showed reduced SWA compared to sham, the fifth PSI following the last stimulation interval showed a rebound increase in SWA (Fig. 4). Potential mechanisms for this rebound may include a homeostatic pressure underlying Slow Wave generation [40] or a reversal of the effect of stimulation from inhibitory to excitatory due to the sustained nature of stimulation [41], [42].

Marshall and colleagues [16] report that intermittent application of tDCS – using the same stimulation duration, frequency and current as in our investigation – enhances slow oscillations during nocturnal sleep and improves memory consolidation. They applied frontal-to-mastoid tDCS (Fig. 1B) to maximally stimulate the whole cortex with slow oscillations originating in the prefrontal cortex [27]. It is not clear though that it was the frequency of the pulsations and not current per se which was responsible for the memory improvement [42], as intermittent tDCS has been reported to have the same effects on neural excitability as constant tDCS [43], [44], particularly when total current over time is matched [45]. We used a cross-hemispheric frontal electrode montage in conjunction with tACS (instead of tDCS) and found this disrupted slow wave generation as expected, suggesting that it is the montage, and not the electric current as such (i.e. mere presence of any exogenous non-zero amperage), that determines the effects of transcranial electrical stimulation on sleep and memory consolidation. The frequency of stimulation was likely an important factor in Marshall and colleagues' study [16], as indicated in a follow up study [46] that reports 5 Hz intermittent tDCS reduced, rather than augmented, slow oscillation power (Though see Ref. [47] for a failure to replicate augmentation with 0.75 Hz stimulation). However, we would expect that with our montage, other frequencies besides what we applied would still disrupt low frequency oscillation owing to the hemispheric asymmetry induced by the cross-hemispheric electrode placement.

A limitation of our study is that there is no inter-hemispherical synchronous stimulation control condition for comparison to our cross-hemispheric stimulation. Further research is warranted to more firmly establish that cross-hemispheric stimulation disrupts SWA because of the inter-hemispheric asynchrony in stimulation; however, the disruption in SWA seen in our study and the boosting of SWA in the study by Marshall and colleagues [16] suggest this mechanism of action and highlights the methodological importance of stimulation montage on the effects of transcranial stimulation. Also, our study had a small sample size, and a larger independent study is warranted to confirm the preliminary effects we report here. Further research on the effects of tACS is also needed as the sustained polarization thought to mediate the effects of tDCS does not occur, and thus the mechanism underlying its observed clinical effects remains to be fully elucidated [29], [48], [49], [50], [51], [52], [53], [54].
