# Supplementary material for: Cross-hemispheric Alternating Current Stimulation During a Nap Disrupts Slow Wave Activity and Associated Memory Consolidation
Source: Brain Stimul. 2015 May-Jun;8(3):520–7. doi: 10.1016/j.brs.2014.12.010 (PMC4464303; doi:10.1016/j.brs.2014.12.010)
Supplement: Supplementary Material [file mmc1.docx]

**Supplementary Methods**

*Psychometric tests*

The Digit Span Test (DST) (1) required subjects to repeat lists of orally presented digits forwards and then backwards. The list started with two digits and increased sequentially by one digit, to a maximum of 9 digits. The experimenter was allowed to repeat each sequence once if necessary. If a mistake was made, the experimenter read a new sequence of the same length. Once two mistakes had been made for the same length sequence, the test was terminated and the results noted.

The Word Fluency Task (1) required subjects to orally name as many words in a given category as they could within a one-minute period. One category was used pre-napping, and another post-napping. In the first experimental session, subjects were required to name types of jobs or hobbies. In the second session subjects were required to list words beginning with the letters ‘M’ or ‘P’. Repeat and incorrect responses were excluded.

*Paired Associate Learning (PAL) task*

Subjects performed a PAL task commonly used to measure declarative memory recall (Figure 1c) (2, 3, 4). The word pairs were nearly identical to the English translations of the German words used in the Born and Marshall (2006) study, which were matched for imeageability, concreteness and frequency. Small alterations were made where translation resulted in nonsense pairs. The task was presented on a computer screen using E-prime software (5). A practice session with only three word pairs was employed to ensure subjects understood the task.

Subjects then underwent training for the experimental task. Instructions appeared on the screen, followed by the presentation of either word pair list version A or B. 54 pairs were presented but only 46 of these were tested. The first and last four pairs of the list were excluded from testing, to control for primacy and recency effects. To prevent order effects, word pairs were presented in random order each time the list was presented. Each word was presented for 1000 ms, with an inter-stimulus interval (ISI) of 300 ms between words in a pair, and 2000 ms between pairs.

Following the initial presentation of the word pairs, subjects were tested without feedback. In each testing trial, a cue word was presented on the screen and subjects were asked to verbally report the corresponding paired word. The experimenter logged their accuracy out of view of the subject and initiated the next cue word. If subjects scored below 30% accuracy, the word pairs were presented again at twice the initial presentation rate, and subjects were re-tested. Note that this 30% accuracy cut-off did not reflect actual performance across subjects on average (see Supplemental Results). After nap, subjects were again presented the cue words and asked to verbally report the pair words.

*Polysomnographic (PSG) Recording*

PSG recordings were collected using 9 mm diameter gold-plated disc electrodes, a Comet XL Lab-based PSG amplifier, and Twin PSG software (Grass-Telefactor, West Warwick, RI, USA) on a PC laptop. Electrodes were placed at International 10:20 System (6) locations O1, C3, C4 and Fz. A1 (left mastoid) served as reference. Additionally, electrodes for recording the EOG were placed 1 cm away from the outer canthus of each eye and 1cm below the centre of the right eye in order to detect vertical and horizontal eye movements. Two electrodes were placed on the chin to monitor submentalis muscular tone. This helped determine if the subject was still sleeping during stimulation periods, as the tACS interfered with all other channels. The skin at each location was cleaned with alcohol and exfoliated with electrolytic abrasive before applying electrodes so as to reduce impedance. A conductive, adhesive, water-soluble wax (Ten20 Conductive Paste by Weaver and Company) was then used to attach each electrode to the subject. The data acquisition rate was 200 Hz, with a high- and low-pass filter set to 0.3 Hz and 35 Hz respectively and a notch filter of 50 Hz. Before lights off, impedance checks were conducted to ensure that impedance values did not exceed 5 kΩ. Subjects were offered earplugs, but their use was not compulsory.

*Analyses on Visually Scored Sleep Data*

Several sleep variables were computed from the visually scored sleep stage data. In order to carry out additional quantitative analyses, non-REM stages were assigned number values of 1, 2, 3, and 4 in each 30-second epoch, besides those with stimulation or major motion artifacts in them.

An analysis conceptually corresponding to the main spectral analyses for each PSI was conducted. Specifically, the number values in the first scored epoch of each PSI minus the pre-stimulation epoch stage value were calculated for each subject. This provides a quantitative index of stage transition from pre-stimulation at each PSI, where positive values correspond to transitions to deeper sleep stages. One-tailed t-tests were conducted to test for significant differences from zero at each PSI and stimulation/sham condition so as to determine when sleep stage in a PSI was significantly different from pre-stimulation on average across subjects.

Proportions of sleep stages following final stimulation or sham intervals (until lights on) were calculated so as to characterize the sleep architecture of the whole sleep opportunity periods following stimulation for both stimulation and sham conditions. Additionally, a similar sleep stage proportion analysis was calculated but with equated numbers of epochs, so as to better characterize potential effects of stimulation/sham conditions on the sleep architecture following the final stimulation/sham interval. The number of epochs in this epoch-equated analysis was 70, the shortest number of epochs following the final stimulation/sham interval across all sessions. Means and averages were calculated across subjects and two-tailed paired t-tests were conducted between stimulation and sham for all of these analyses.

**Supplemental Results**

*Pre-nap Paired Associate Learning (PAL) task performance*

Across all sessions the mean PAL task performance before nap was 53.26% (about 24.5 word pairs) with a standard deviation of 16.68%.

*Sleep Variables*

Sleep Variables based on visual scoring criteria are reported for the sham condition in Supplementary Table 1. Sleep latency variables for both stimulation and sham conditions are reported in Supplementary Table 2.

*Change in Sleep Depth from Pre-Stimulation Interval at each PSI*

Analyses on indices of sleep stage transition from pre-stimulation at each PSI (see Supplementary Methods; Supplementary Figure 1A) revealed that for the sham condition, subjects were on average in deeper sleep stages compared to the pre-sham epoch significantly for PSI 1 (p < 0.045, two-tailed). Sham PSIs 2 through 5 revealed only numerically deeper sleep, but not significantly deeper (all p > 0.09, two-tailed). Stimulation condition PSIs revealed marginally significant deeper sleep compared to pre-stimulation significant for PSIs 3, 4, and 5 (all p < 0.062, two-tailed). PSIs 1 and 2 for the stimulation condition revealed no significant differences in sleep stage depth from pre-stimulation. Comparisons between stimulation and sham conditions at each PSI did not reveal significant differences (all p > 0.11, two-tailed). Comparing these analyses with the corresponding spectral analyses on Slow and Delta power, a qualitative correspondence can be seen in PSI’s 1 and 5, where transition to deeper sleep is suppresed in PSI 1 for stimulation but not sham and a numerical greater sleep depth for stimulation relative to sham is seen in PSI 5. The unclear correspondence between the two analysis types in PSIs 2 through 4 likely reflects that spectral analysis is a more sensitive measure of SWA difference than visual scoring because it is a continuous rather than discrete measure.

*Sleep Stage Proportions Following Final Stimulation*

There was no significant difference between stimulation and sham conditions in time following the final stimulation/sham interval until lights on (stimulation: 3394 ± 899 seconds; sham: 3004 ± 792 seconds; p > 0.35, two-tailed). Proportions of sleep stages following final stimulation or sham intervals until lights on (Supplementary Figure 1B) revealed no significant differences between stimulation and sham conditions for any sleep stage (all p < 0.17, two-tailed), except for stage 4 (p < 0.040, two-tailed) though this difference does not survive Bonferroni correction. Proportions of sleep stages following final stimulation but limited to until 70 epochs had passed (Supplementary Figure 1C) also revealed no significant differences between stimulation and sham conditions for any sleep stage (all p < 0.092, two-tailed), except for stage 3 (p < 0.040, two-tailed) though this difference also does not survive Bonferroni correction.

**Supplementary Information Reference List**

1. Wechsler, D. (2008). *Wechsler Adult Intelligence Scale–Fourth Edition(WAIS–IV)*.San Antonio, TX: Pearson.
2. Plihal W, Born J (1997) Effects of Early and Late Nocturnal Sleep on Declarative and Procedural Memory. J. Cogn. Neurosci. 534-547.
3. Marshall L, Molle M, Hallschmid M, Born J (2004) Transcranial Direct Current Stimulation During Sleep Improves Declarative Memory. J. Neurosci. 9985-9992.
4. Marshall L, Helgadottir H, Molle M, Born J (2006) Boosting Slow Oscillations During Sleep Potentiates Memory. Nature 610-613.
5. Schneider, W., Eschman, A., & Zuccolotto, A. (2002) E-Prime Reference Guide. Pittsburgh: Psychology Software Tools Inc.
6. Jasper H (1958) The Ten-Twenty Electrode System of the International Federation. EEG Clin. Neurophysiol. 371-375.

Supplementary Figure Legends

**Supplementary Figure 1. Sleep Stage Scoring Analyses at each PSI and during Post-Stimulation Sleep Opportunity.** Sleep stages were determined through visual scoring. A. Change in sleep stage depth in each PSI (See Supplementary Methods). B. Proportion of sleep stages in the sleep opportunity from final stimulation interval until lights on. C. Proportion of sleep stages in the sleep opportunity following the final stimulation but truncated at 70-epochs post-stimulation so as to equate the time of interest across all experimental sessions.

**Supplementary Table 1. Sleep Variables for Sham Condition Based on Visual Sleep Stage Scoring.** Means (± Standard Deviations).

**Supplementary Table 2. Sleep Latencies for Stimulation and Sham Conditions Based on Visual Sleep Stage Scoring.** Means (± Standard Deviations). Epochs are 30 seconds each. “Sleep onset” is defined by the occurrence of three contiguous epochs of sleep. One subject in the stimulation session entered Stage 2 before entering Stage 1, hence the non-zero latency for sleep onset to Stage 2.
