# Supplementary material for: Cross-hemispheric Alternating Current Stimulation During a Nap Disrupts Slow Wave Activity and Associated Memory Consolidation
Source: Brain Stimul. 2015 May-Jun;8(3):520–7. doi: 10.1016/j.brs.2014.12.010 (PMC4464303; doi:10.1016/j.brs.2014.12.010)
Supplement: Supplementary Figure 1 [file mmc2.docx]

A

Supplementary Figure 1

B

C
