# Supplementary material for: Cross-hemispheric Alternating Current Stimulation During a Nap Disrupts Slow Wave Activity and Associated Memory Consolidation
Source: Brain Stimul. 2015 May-Jun;8(3):520–7. doi: 10.1016/j.brs.2014.12.010 (PMC4464303; doi:10.1016/j.brs.2014.12.010)
Supplement: Supplementary Tables 1 and 2 [file mmc3.docx]

| **Sleep Variables** | **Number of Epochs** |
| --- | --- |
| Total Dark Time | 235.7 (±49.3) |
| Sleep Period | 207.4 (±51.6) |
| Total sleep time | 186 (±57.9) |
| Sleep before sleep onset | 2.2 (±1.8) |
| Wake after sleep onset | 11.2 (±15.5) |
| Wake after final awakening | 1.7 (±4.5) |
| Total wake time | 31 (±48) |
| Stage 1 | 80.7 (±26.8) |
| Stage 2 | 74.5 (±52.6) |
| Stage 3 | 15 (±14) |
| Stage 4 | 15.7 (±19) |
| REM | 0 (±0) |
| MT | 10.1 (±6.4) |
| NREM | 186 (±57.9) |
| SW | 30.7 (±32) |

|  | **Number of Epochs** | |  |
| --- | --- | --- | --- |
| **Variable** | **Stim** | **Sham** | **Significance of Difference** |
| Start to Sleep Onset | 10 (±7.2) | 19.2 (±30.2) | 0.35 |
| Sleep onset to Stage 1 | 18.7 (±49.5) | 0 (±0) | 0.35 |
| Sleep onset to Stage 2 | 27 (±42.7) | 20.1 (±27.8) | 0.73 |
| Sleep onset to Stage 3 | N/A | 23.6 (±14.7) | N/A |
| Sleep onset to Stage 4 | N/A | 41.2 (±13.9) | N/A |
| Sleep onset to REM | N/A | N/A | N/A |

Table 2: Sleep Latencies – Stim and Sham

Table 1: Sham Sleep Variables

Supplementary Tables
